# Wavelength-tunable sources of entangled photons interfaced with atomic vapours

**DOI:** 10.1038/ncomms10375

**Published:** 2016-01-27

**Authors:** Rinaldo Trotta, Javier Martín-Sánchez, Johannes S. Wildmann, Giovanni Piredda, Marcus Reindl, Christian Schimpf, Eugenio Zallo, Sandra Stroj, Johannes Edlinger, Armando Rastelli

**Affiliations:** 1 Institute of Semiconductor and Solid State Physics, Johannes Kepler University Linz, Altenbergerstr. 69, A-4040 Linz, Austria; 2 Forschungszentrum Mikrotechnik, FH Vorarlberg, Hochschulstr. 1, A-6850 Dornbirn, Austria; 3 Institute for Integrative Nanosciences, IFW Dresden, Helmholtzstr. 20, D-01069 Dresden, Germany; 4 Paul-Drude-Institut für Festkörperelektronik, Hausvogteilplatz 5-7, 10117 Berlin, Germany

## Abstract

The prospect of using the quantum nature of light for secure communication keeps spurring the search and investigation of suitable sources of entangled photons. A single semiconductor quantum dot is one of the most attractive, as it can generate indistinguishable entangled photons deterministically and is compatible with current photonic-integration technologies. However, the lack of control over the energy of the entangled photons is hampering the exploitation of dissimilar quantum dots in protocols requiring the teleportation of quantum entanglement over remote locations. Here we introduce quantum dot-based sources of polarization-entangled photons whose energy can be tuned via three-directional strain engineering without degrading the degree of entanglement of the photon pairs. As a test-bench for quantum communication, we interface quantum dots with clouds of atomic vapours, and we demonstrate slow-entangled photons from a single quantum emitter. These results pave the way towards the implementation of hybrid quantum networks where entanglement is distributed among distant parties using optoelectronic devices.

## Abstract (web summary)

Quantum dots can produce entangled photons, but careful control over photon wavelength is required for real-life applications. Here, the authors use three-directional strain engineering to overcome this problem and to interface entangled photons from quantum dots with a cloud of natural atoms.

## Graphical abstract

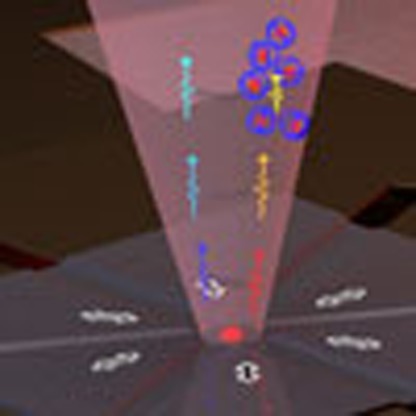

The possibility of exploiting quantum effects in the solid state for quantum communication applications is nowadays one of the main driving forces behind current research efforts on semiconductor nanostructures. While the investigation of single nanostructures as hosts or sources of quantum bits is already advanced, using several of them for the envisioned applications requires an important challenge to be overcome: nanostructures are not identical and their properties cannot be predicted with the desired accuracy, a property arising from our limited capability to control their fabrication processes with atomic-scale precision^1^. A prominent example is represented by semiconductor quantum dots (QDs)^2^. During the radiative decay of a confined biexciton XX, these nanostructures can generate entangled photon pairs^3^,^4^,^5^,^6^ with high efficiency^7^,^8^,^9^, high degree of entanglement^9^,^10^,^11^, high indistinguishability^12^,^13^, and—in contrast to parametric down-conversion^14^ and four-wave mixing^15^ sources—they can deliver photons deterministically^12^,^16^. Compared with other single quantum emitters^17^, QDs have the advantage of being compatible with the mature semiconductor technology^18^,^19^. For these reasons, it has been recently argued that they have the potential to become the ‘perfect' sources of entangled photons^20^. In spite of these accomplishments, there are two points that are often overlooked: First, in the presence of unavoidable structural asymmetries the anisotropic electron-hole exchange interaction induces a fine structure splitting (FSS; *s*) between the intermediate exciton X levels^21^ that markedly lowers the degree of entanglement of the source^22^, which eventually emits only classically polarization-correlated photons. Even though methods to suppress the FSS exist^6^,^23^, advanced quantum optics experiments^24^ are still carried out using single ‘hero' QDs that have—for probabilistic reasons^25^—zero FSS. Second, even having at hand a bunch of these special QDs, each of them emits entangled photons at a different random energy, and any attempt to modify these energies via external perturbations restores the FSS, thus spoiling entanglement^10^. This represents a serious hurdle when one aims at using several QDs for quantum communication. In fact, the tunability in energy of the entangled photons is a fundamental prerequisite to teleport entanglement between the distant nodes of a quantum network via quantum interference^26^ and, to date, has been demonstrated only for Poissonian sources of entangled photons^27^,^28^. Control over the photon energy is also an essential requirement for efficient photon storage in quantum memories based on atomic clouds^29^ or Bose–Einstein condensates^30^, as the existing protocols require precise colour matching with atomic resonances.

Here we demonstrate that it is possible to control the energy of the entangled photons emitted by arbitrarily selected QDs without degrading the degree of entanglement of the quantum source. The core idea of our work is to manipulate the strain state of the QD and surrounding semiconductor matrix so as to achieve full control over the anisotropic electron-hole exchange interaction^21^, and to modify the energy levels involved in the generation of entangled photons (X and XX) without opening the FSS. Addressing this task is not trivial. Theory^6^,^23^ and experiments^6^ have demonstrated that the X level degeneracy of any arbitrary QD can be restored via the combined action of two independent external perturbations, such as stress and electric fields. Suppression of the FSS occurs, however, for a particular combination of the magnitude of the two fields and, as a consequence, for a rather small and unpredictable spectral range of the emitted photons^10^. Although for QDs with special properties energy tuning at small (albeit non zero) FSS can be achieved using a combination of magnetic and electric fields^31^, the demonstration of a truly energy-tunable source of entangled photons—whose entanglement degree is not dependent on the photon energy—has been till now lacking. Recently, some of us have theoretically shown that an energy-tunable source of entangled photons based on an arbitrary QD requires at least three independent ‘tuning knobs'^32^, thus explaining the unsuitability of previous approaches^10^,^31^. Such degrees of freedom are conveniently provided by the three components of the QD in-plane strain tensor. We now present the experimental implementation of this theoretical concept. To benchmark our results we precisely tune a QD (‘artificial atom') to emit entangled photons in the spectral region between double absorption resonances of natural atoms, and we demonstrate slow-entangled photons.

## Results

### Mastering the exciton fine structure splitting

**Figure 1:**
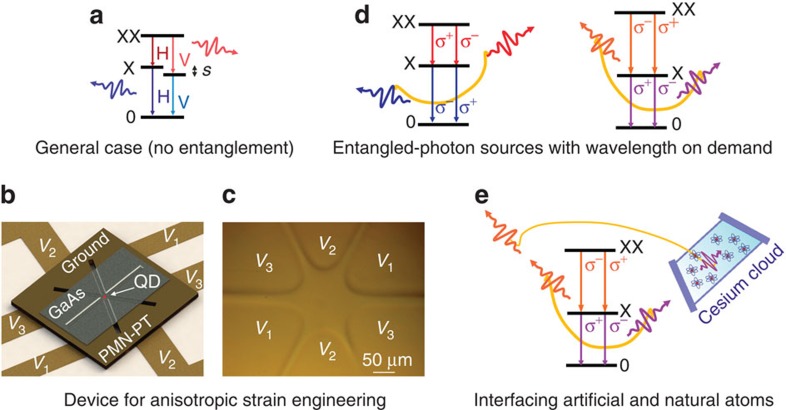
A six-legged semiconductor-piezoelectric device for quantum optics. (**a**) Sketch of the radiative decay of a confined biexciton (XX) to the ground state (0) in a generic as-grown QD. In the presence of a FSS (*s*), the emitted photons are only classically correlated. H (V) indicates horizontally (vertically) polarized photons. (**b**) Sketch of the six-legged device used to engineer the strain status of a nanomembrane (grey region) containing QDs. (**c**) Microscope picture of the central part of the final device. (**d**) Same as in **a** for a QD embedded in the device show in **c**, where anisotropic in-plane strains are first used to restore the exciton (X) degeneracy (left panel), and then to modify the X and XX energies without affecting the FSS (right panel). The yellow lines indicate entanglement. σ^+^ (σ^−^) indicates right (left) circularly polarized photons. (**e**) Sketch of a QD whose X photon—polarization entangled with the XX photon—is tuned to the middle of the hyperfine levels of cesium (Cs) and is slowed down.

Our device (Fig. 1b,c) is built-up merging piezoelectric and semiconductor technologies^33^and it allows any arbitrary QD (Fig. 1a) to be tuned for the generation of polarization-entangled photons with tunable wavelength (Fig. 1d) in the spectral region of the D_1_ lines of a cloud of cesium atoms (Fig. 1e). The self-assembled In(Ga)As QDs studied here are embedded in the free-standing area of a 300-nm-thick GaAs nanomembrane that is bonded onto a micromachined 300-μm-thick [Pb(Mg_1/3_Nb_2/3_)O_3_]_0.72_-[PbTiO_3_]_0.28_ (PMN-PT) piezoelectric substrate. The actuator features six trapezoidal areas (‘legs') separated by trenches aligned at ∼60° with respect to each other (for details about the device fabrication, see Supplementary Fig. 1 and Supplementary Note 1). The key idea behind this design is that full control of the in-plane strain tensor can be achieved by applying three independent uniaxial stresses in the nanomembrane plane. With our actuator, quasi-uniaxial stresses in the membrane can be obtained by applying three independent voltages (*V*_1_, *V*_2_ and *V*_3_) at the bottom of opposite legs (labelled as Leg 1,2,3) with respect to the top part of the piezo, which is electrically grounded. Figure 1c shows a top-view microscope picture of the central part of the device. The regions of the nanomembrane that are suspended can be clearly distinguished from those that are bonded on the piezo legs.

**Figure 2:**
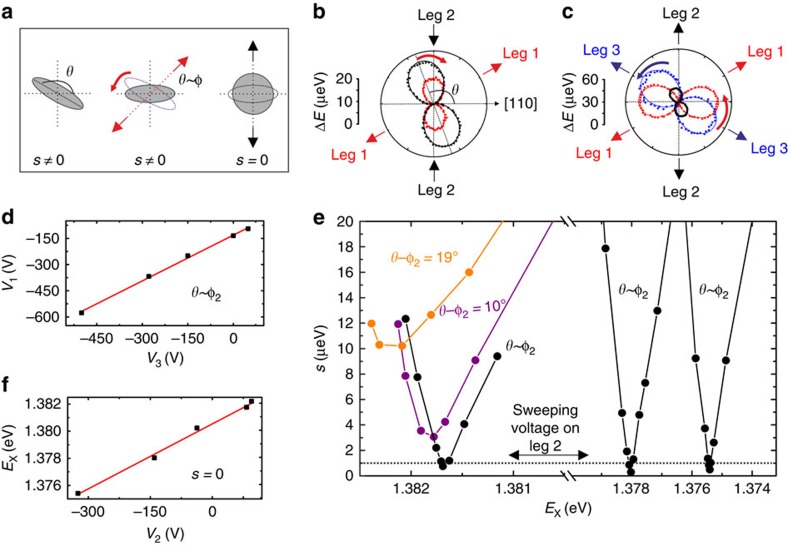
Tuning the exciton energy at zero fine structure splitting. (**a**) Sketches of the in-plane QD anisotropy under the effect of external perturbations, see the main text. (**b**) Dependence of Δ*E* (see Methods) as a function of the angle the linear polarization analyser forms with the [110] crystal axis. The length and orientation of the petals give the value of the *s* and *θ*, respectively. The black data points correspond to zero applied voltages, while the red data points show the configuration in which Leg 1 is used to achieve *θ*∼φ_2_. The solid lines are sinusoidal fits to the experimental data. (**c**) Same as in **b** when Leg 3 is used to change the QD strain status (see the blue data points) while Leg 1 is again used to achieve *θ*∼φ_2_ (see the red data points). The curve for zero applied voltages is also reported for reference (see the black data points). (**d**) Linear dependence of *V*_1_ versus *V*_3_ when *θ*∼ φ_2_. (**e**) Behaviour of the FSS as a function of *E*_X_, modified as described in the text. The dashed line shows the threshold of 1 μeV. (**f**) Behaviour of *E*_X_ as a function of *V*_2_ at *s*=0.

The first question we address is: how to achieve full control over the FSS of any arbitrary QD? External fields with two different degrees of freedom are required to master two different QD parameters^32^: the magnitude of the FSS (*s*), and the polarization direction of the exciton emission (*θ*). The latter parameter gives the in-plane orientation of the exciton dipoles with respect to the crystal axis^34^, and it provides information about the QD anisotropy that is fundamental to drive the device during the experiment. A robust approach^6^ to achieve zero FSS in any QD with two ‘tuning knobs' can be illustrated by picturing the QD anisotropy as an ellipse with axes given by the two in-plane spin–spin coupling constants^21^ (Fig. 2a): the FSS can be suppressed every time one external field is used to align *θ* along the direction of application of the second field (*φ*, see the central panel of Fig. 2a), which is then capable to compensate completely for the asymmetries in the in-plane QD confining potential (see the right panel of Fig. 2a), that is, it is capable to tune the FSS through zero. Following this picture, the first step we performed in the experiment is a polarization-resolved measurement aimed at quantifying *s* and *θ* for a randomly chosen QD when no voltages are applied to the piezo legs. These two quantities are encoded in the polar plots of Fig. 2b, where the length and orientation of the ‘petals' give the value of the *s* and *θ*, respectively (see Methods). The measurement at zero applied voltages (see the black data points in Fig. 2b) reveals *s*=20±0.3 μeV and *θ*=109°±0.4° with respect to the [110] direction of the GaAs nanomembrane, which was aligned perpendicularly to Leg 2 during device fabrication. Since *θ* differs from the direction of application of the stress exerted by Leg 2 (labelled φ_2_ in the figure) by 19°, it is not possible to suppress the FSS using only this leg. In fact, Fig. 2e shows clearly that sweeping the voltage across Leg 2 leads to an anticrossing between the two X states^35^, with a lower bound for the FSS of ∼10 μeV. The X degeneracy can instead be restored by first using Leg 1 to align *θ* along Leg 2 (see red data points in Fig. 2b), and then employing Leg 2 to cancel the FSS. Figure 2e shows that when *θ*∼φ_2_ the FSS can be tuned well below the threshold of 1 μeV usually required to observe entangled photon emission (see the black data points on the left-hand side). These results represent the first experimental evidence that the FSS can be fully controlled with two independent stresses, in line with earlier theoretical predictions^23^. We observe this behaviour in all eight QDs we selected randomly in two different devices, thus proving the general relevance of our findings. It is important to note that, for each QD, there is only one combination of *V*_1_ and *V*_2_ that allow the condition *s*=0 to be reached, similarly to the case of strain and electric field^10^.

The second question is: how to control the X energy (*E*_x_) of an arbitrary QD without re-opening the FSS? In other words: how to use any QD as energy-tunable source of entangled photons? As recently proposed, this can be achieved by adding an isotropic biaxial strain in the plane of the QD^32^,^36^, which does not affect its in-plane anisotropy (Fig. 2a). To accomplish this task we make use of the third pair of piezo legs: By applying a voltage *V*_3_ across it we modify the strain configuration and thus the initial values of *s* and *θ* (see the blue data points in Fig. 2c). We then follow the two-step procedure described above to erase the FSS: (1) We use Leg 1 to rotate *θ* until the exciton aligns along Leg 2 or the perpendicular direction, that is, until we reach the condition *θ*∼φ_2_ (see the Supplementary Fig. 2 and the Supplementary Note 2). The specific direction is determined by the handedness of *θ* under the stress exerted by Leg 1, being clockwise (anticlockwise) when it is larger (smaller) than 120°. (2) Finally, we suppress the FSS by sweeping again the voltage on Leg 2 (Fig. 2e). Having modified the QD strain status with Leg 3, the condition *s*=0 is now obtained for a different combination of *V*_1_ and *V*_2_ and, as a consequence, for a different X energy (see the black data points on the right-hand side of Fig. 2e). It is worth mentioning that the role of the legs can be exchanged without affecting the final results, as there exists only one combination of the three in-plane components of the stress tensor which leads to *s*=0 at each specific X energy^32^. To change *E*_X_, it is then sufficient to repeat the three step procedure described for a different combination of *V*_1_, *V*_2_ and *V*_3_ (see the other black points on the right-hand side of Fig. 2e). This is made easier considering that the voltages *V*_1_ and *V*_3_ to be applied to maintain the condition *θ*∼φ_2_ scale linearly (Fig. 2d). Finally, Fig. 2f shows that at *s*=0 *E*_X_ changes linearly with *V*_2_ and, most importantly, that it can be tuned across a spectral range of ∼7 meV. The experimental results shown so far are fully in line with a theoretical model based on **k̇p** theory that describes the behaviour of the X states under the influence of in-plane strains with variable magnitude and anisotropy^32^. This model further confirms that our six-legged device is capable of delivering three independent stresses to single QDs (Supplementary Fig. 2), the key ingredient to reach the results shown in this work.

### Entangled photon sources with wavelength on demand

**Figure 3:**
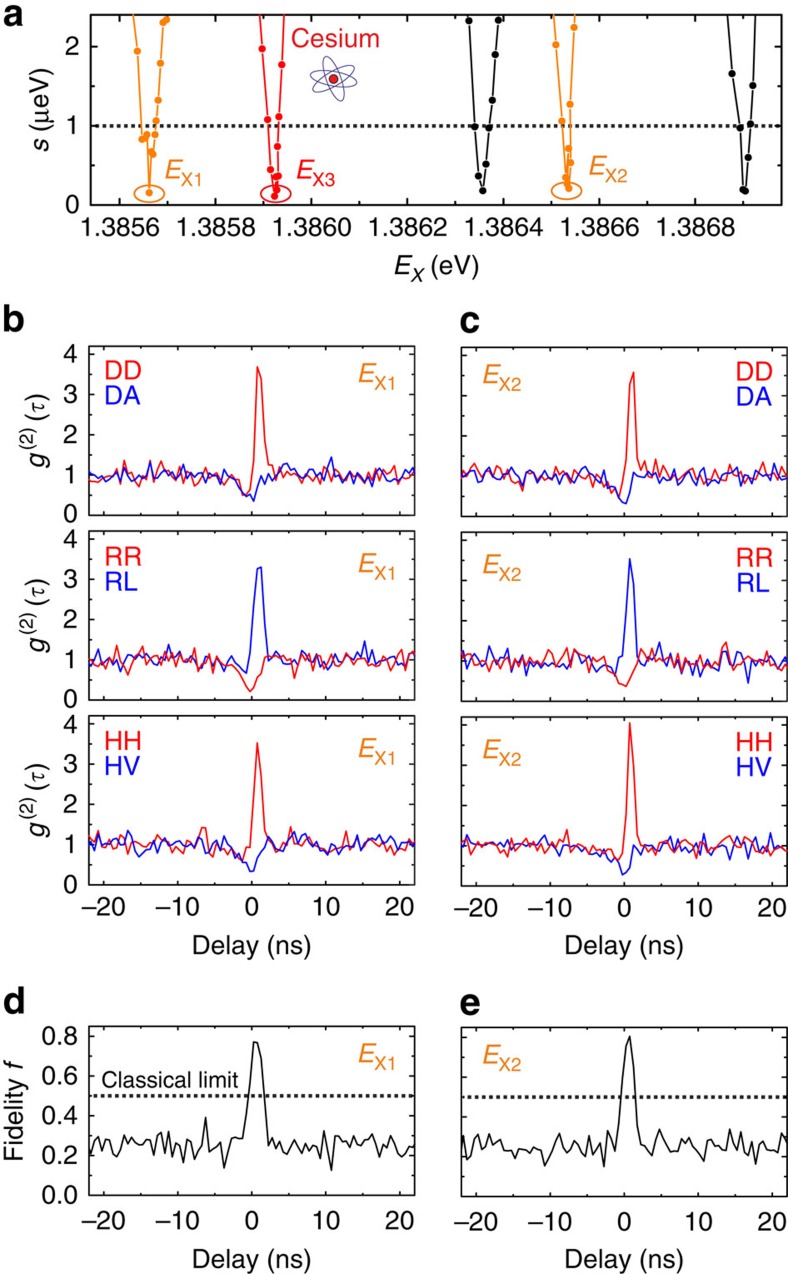
Entangled photon pairs at different photon energies. (**a**) Same as in Fig. 2e for a different QD whose FSS is fine-tuned to *s*<0.2 μeV. The circles indicate the X energies (*E*_X1,2,3_) where cross-correlation measurements have been performed. In particular, *E*_X3_ correspond to the position in between the hyperfine lines of Cs. (**b**) XX-X cross-correlation measurements for diagonal (top panel), circular (central panel) and linear (bottom panel) polarization basis when the X energy is set to *E*_X1_. (**c**) Same as in **b** for *E*_X2_. (**d**) Fidelity as a function of the time delay when the X energy is set to *E*_X1_. The dashed line indicates the threshold for entanglement, that is, the classical limit. (**e**) Same as in **d** for *E*_X2_.

Having demonstrated for the first time that it is possible to tune the energy of the X at *s*=0, we now demonstrate that our device can be used as a truly energy-tunable source of entangled photons, that is, a source where the level of entanglement does not depend on the energy of the emitted photons. For this experiment, we choose a different QD and we repeat the procedure detailed above by carefully tuning the FSS down to zero with a resolution of ∼0.2 μeV (Fig. 3a). For two different X energies (highlighted with *E*_X1_ and *E*_X2_ in Fig. 3a) we performed polarization-resolved cross-correlation measurements between the X and XX photons (see Methods). In the case *s*=0, the two-photon state can be expressed by the maximally entangled Bell state 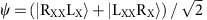, which can be equivalently rewritten as 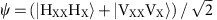 or 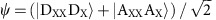, where H (V), D (A) and R (L) indicate horizontally (vertically) polarized, diagonally (antidiagonally) polarized, and right (left)-circularly polarized photons, respectively. Therefore, when performing polarization-resolved cross-correlation measurements, one expects a strong bunching peak for co-linear, co-diagonal, and cross-circular polarization and antibunching for the opposite polarization settings. This is exactly the behaviour we observe in our measurements (Fig. 3b-3c), which represent a clear signature of entanglement. To estimate the degree of entanglement, we calculated the fidelity *f* to the Bell state *ψ* (see Methods) and we found—by integrating over 0.5 ns around the central peak—*f*_1_=0.80±0.05 and *f*_2_=0.78±0.06 for *E*_X1_ and *E*_X2_, respectively (Fig. 3d,e), similarly to what we measured in different QDs by full reconstruction of the two-photon density matrix^10^. These values are larger than the classical limit (0.5) for a source emitting polarization-correlated photons, and prove for the first time that QDs can produce entangled states of light at different energies. The fact that the two values of the fidelity are identical within the error bounds demonstrates that the degree of entanglement of the source does not depend on the photon energy, an important requirement for applications^37^. Despite the fidelity we measure is among the highest ever reported so far for QDs^9^,^10^, it is not yet perfect (∼1) due to depolarization of X states^38^ and recapture processes^7^. However, we strongly believe that the level of entanglement can be even improved further using faster photon detectors and resonant two-photon excitation schemes^12^,^16^.

### Slowing-down entangled photons from QDs

To prove that we are able to control the energy of the entangled photons with the precision required for advanced quantum optics experiments, we show that it is possible to interface entangled photons emitted by a QD with clouds of natural atoms operated as a slow-light medium. Pioneering works on the field^39^,^40^ have demonstrated that single photons emitted by GaAs QDs can be slowed down using warm rubidium vapours, which can be also exploited as absolute energy reference to interconnect the different nodes of a quantum network. The concept has been recently adopted to interface single photons emitted by single molecules with vapours of alkali atoms^41^. However, it has never been applied to slow down entangled photon pairs from single quantum dots. The reason is that the energy of one photon of the entangled pair must match the spectral window of double absorption resonances in warm atomic vapours, and this possibility was out of reach before our work.

**Figure 4:**
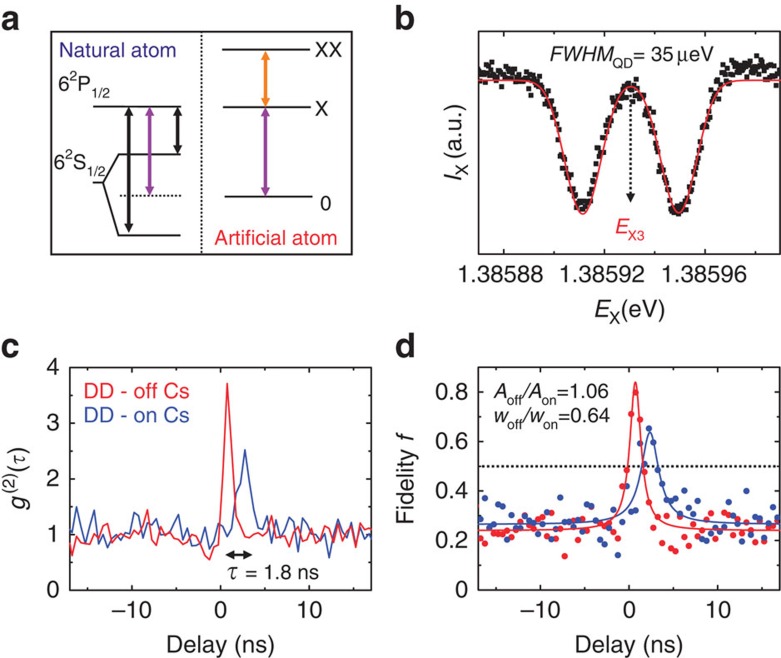
Entangled photons from artificial atoms interfaced with natural atoms. (**a**) Sketch of the energy levels of a Cs atom (left panel) and of a QD fine-tuned for entanglement (right panel). The energy axis (not to scale) has been shifted so that *E*_X_ (purple arrow) matches the middle of the two D_1_ lines of Cs (black arrows). (**b**) Intensity of the X when its energy is swept across the D_1_ lines of Cs. The solid line is a fit to the theoretical model (see Supplementary Note 3) used to extract the linewidth of the X transition (indicated with *FWHM*_QD_). The dashed arrow indicates the *E*_X3_. (**c**) XX-X cross-correlation measurements with the same diagonal polarization with (blue line) and without (red line) the Cs cell in the X optical path. The temporal delay introduced by the Cs cell (*τ*) is also indicated. (**d**) Fidelity with (blue data points) and without (red data points) the Cs cell in the X optical path. The solid lines are Lorentzian fits to the data. The ratio between the full-width-half maxima (FWHM; *w*) of the curves and the integrated area (*A*) are also indicated.

The emission spectrum of our QDs is centred at ∼900 nm, close to the D_1_ lines of cesium (Cs). Therefore, we insert a temperature-stabilized quartz cell containing Cs vapour^42^,^43^ in the optical path of the exciton photon (see Methods). Figure 4a shows a sketch of the Cs D_1_ lines of relevance for this experiment, that is, the transitions involving the 6^2^P_1/2_ level and the hyperfine-split 6^2^S_1/2_ doublet^44^. To observe slow-entangled photons, one has to tune the energy of the X (or XX) transition in between the hyperfine lines^39^ while *s* is kept at 0 (see purple arrows in Fig. 4a). Experimentally, this condition is identified by recording the intensity of the X emission while scanning its energy across the Cs lines (Fig. 4b). This allows us to observe two absorption resonances resulting from a convolution between the inhomogeneously broadened X transition and the Doppler-broadened Cs doublet (Supplementary Note 3). When the X energy is tuned exactly in the middle of the hyperfine lines (*E*_X3_, Figs 3a and 4b), the QD photons probe a strongly dispersive medium and are slowed down^39^. Taking into account the temperature of the Cs cell used in the experiment (*T*_Cs_=140 °C), we estimate a differential delay of up to ∼2 ns on a 7.5 cm long path. To observe this delay, it is sufficient to perform X-XX cross-correlation measurements with and without the Cs cell. Figure 4c shows the result of such measurements when both X and XX photons are collected with the same diagonal polarization. The bunching peak clearly shifts by >1.8 ns when the Cs cell is inserted into the optical path. However, the peak appears less pronounced and broader compared with the measurements performed without Cs cell, which we attribute to dispersion combined with the spectral broadening of the X emission line (∼35 μeV, see Fig. 4b, Supplementary Note 3 and ref. 43). It is therefore interesting to investigate whether the degree of entanglement of photon pairs is affected by the presence of the atomic cloud. Figure 4d shows the fidelity calculated after performing XX-X cross-correlation measurements with the same polarization settings used in Fig. 3b-3c. The measurements without Cs cell show a peak fidelity of *f*_3_^off^=0.8±0.02, which nicely matches the values obtained for the other *E*_X1_ and *E*_X2_ energies (Fig. 3) and further confirms that the degree of entanglement of our source does not change with the X energy. The measurements with the Cs cell show instead a peak fidelity of *f*_3_^on^=0.65±0.08. Despite this value is above the classical limit of 0.5, it may suggest a degradation of the degree of entanglement. However, careful inspection of the data shows that this is not the case and that the peak fidelity is not the most accurate parameter to estimate entanglement. The solid lines in Fig. 4d show lorentzian fits to the experimental data. In the presence of the Cs cloud there is a considerable temporal broadening (a factor almost 2), while the integrated area (*A*) remains practically unchanged (*A*_off_/*A*_on_=1.06). These results clearly imply that the degree of entanglement of the photons is not affected by the presence of the Cs cell, thus opening new venues for advanced quantum optics experiments with the hybrid artificial–natural atomic interface.

## Discussion

In conclusion, we have demonstrated that it is possible to modify the energy of the polarization-entangled photons emitted by arbitrary QDs without affecting the degree of entanglement of the photons. These results have been achieved by developing a novel class of electrically controlled semiconductor-piezoelectric devices that allow the optical and electronic properties of every single QD to be arbitrarily re-shaped via anisotropic strain engineering. The tunability of the photon energy has opened up the unprecedented possibility to interface entangled photons from QDs with clouds of natural atoms and, in turn, to demonstrate for the first time slow-entangled photons from a single QD. In light of our results, it is possible to envisage a new era for QDs in the field of large distance quantum communication. In fact, dissimilar QDs can now be used for entanglement teleportation (or swapping), as the entangled photons can be colour matched for quantum interference at a beam splitter^26^. Furthermore, the hybrid artificial-atomic interface we have built-up can be further developed up to the limit where entangled states are stored and retrieved at the single-photon level^29^. This could allow the whole concept of a quantum repeater^45^ to be implemented. Addressing these applications most likely requires the use of our energy-tunable entangled-photon source in combination with broad-band photonic structures featuring GaAs microlenses that are capable to boost the flux of QD photons^46^,^47^ and with resonant excitation schemes for the deterministic generation of entangled photons with high degree of indistinguishability^12^,^16^. However, the implementation of the perfect source of entangled photons is worth the efforts, as the realization of a solid state-based quantum network for the distribution of quantum entanglement among distant parties^48^ will be revolutionary.

## Methods

### Sample growth and device fabrication

In(Ga)As QDs were grown by molecular beam epitaxy. Following oxide desorption and buffer growth, a 100-nm-thick Al_0.75_Ga_0.25_As sacrificial layer was deposited before a 300-nm-thick GaAs layer containing the QDs. The QDs were grown at 500 °C and capped by an indium flush technique. Using standard epoxy-based photoresist, the sample (coated with a 100-nm-thick Cr/Au layer) was integrated via a flip-chip process onto a Cr/Au-coated micro-machined PMN-PT actuator. The GaAs substrate, buffer layer and AlGaAs sacrificial layer were removed using wet chemical etching (Supplementary Fig. 1), thus leaving the GaAs nanomembrane tightly bound on the piezoelectric actuator. The PMN-PT actuator was processed in the six-leg design shown in Fig. 1 with a femtosecond laser having central wavelength of 520 nm, pulse duration of 350 fs, repetition rate of 25 kHz and a ∼5 μm spot size. Six metal contacts were fabricated at the bottom of the piezo legs so that independent voltages can be applied with respect to the top contact, which is set to ground. The same voltage is applied to opposite legs to limit displacements of the central structure. The voltage applied on each pair of legs leads to a well-controlled deformation of the GaAs nanomembrane suspended in the central part (Supplementary Note 4 and Supplementary Fig. 3). The piezoelectric actuator was poled so that a voltage *V*<0 (*V*>0) applied to each pairs of aligned legs result in an out-of-plane electric field that leads to an in-plane contraction (expansion) of the piezo leg. Therefore, an in-plane tensile (compressive) strain is transferred to the central part of the nanomembrane (Supplementary material). In the tensile regime (*V*<0), the X emission line of the QD of Fig. 2 shifts approximately linearly with the applied voltages, with a slope that depends on the used leg. In particular, we measure slopes of 10, 7, 3 μeV/V for Legs 1, 2 and 3, respectively. This difference may arise from the fact that the QD under investigation is not sitting in the very central part of the device (Supplementary Note 4).

### Micro-photoluminescence and photon-correlation spectroscopy

Conventional micro-photoluminescence spectroscopy is used for the optical characterization of the devices. The measurements are performed at low temperatures (typically 4–10 K) in a helium flow cryostat. The QDs are excited non-resonantly at 840 nm with a femtosecond Ti:Sapphire laser and focused by a microscope objective with 0.42 numerical aperture. The measurements in Figs 3 and 4 appear to be performed with a continuous wave laser due to long lifetime of the X transition. The same objective is used for the collection of the photoluminescence signal, which is spectrally analysed by a spectrometer and detected by a nitrogen-cooled silicon charge-coupled device. Polarization-resolved micro-photoluminescence experiments are performed combining a rotating half-wave plate and a fixed linear polarizer placed after the microscope objective. The transmission axis of the polarizer is set parallel to the [110] direction of the GaAs crystal (within 2°) and perpendicular to the entrance slit of the spectrometer, which defines the laboratory reference for vertical polarization. The FSS and the polarization angle of the X emission are evaluated using the same procedure reported in ref. 10, which ensures sub-microelectronvolt resolution. In particular, Fig. 2 shows Δ*E* as a function of the angle the linear polarization analyser forms with the [110] crystal axis, where Δ*E* is half of the difference between the XX and X energy minus its minimum value.

For photon-correlation measurements, the signal is split into two parts after the microscope objective using a non-polarizing 50/50 beam splitter, spectrally filtered with two independent spectrometers tuned to the XX and X energies, and finally sent to two Hanbury Brown and Twiss setups. Each Hanbury Brown and Twiss setups consists of a polarizing 50/50 beam splitter placed in front of two avalanche photodiodes (APDs), whose output is connected to a four-channel correlation electronics for reconstructing the second-order cross-correlation function between the XX and X photons, *g*^(2)^(*τ*). The temporal resolution of the set-up is ∼500 ps, mainly limited by the time jitter of the APDs. Properly oriented half-wave plates and quarter-wave plates are placed right after the non-polarizing beamsplitter to select the desired polarization. With a single measurement, this experimental set-up allows 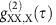 to be reconstructed in four different polarization settings, so as to minimize the effect of possible sample drifts. The count rate at each APD is typically 10^3^–10^4^ count per seconds and the integration time used for each measurement is ∼1 h.

For cross-correlation measurements in the presence of the atomic cloud we use a 7.5-cm-long quartz cell filled with Cs vapours and wounded up with heating foils for precise temperature tuning. The temperature chosen for the experiment is 140±0.1 °C. The Cs cell is inserted in the X optical path, after the non-polarizing beam splitter and the retarder wave plates, right before the entrance slit of the spectrometer. When the X is tuned in between the hyperfine lines of Cs, the count rate at the APDs is reduced by a factor of ∼2 due to the optical absorption in the Cs vapour.

### Entanglement analysis

Raw data were used to evaluate the second-order correlation functions, without any background light subtraction. As mentioned in the previous section, the experimental set-up allows the second order XX-X cross-correlation function 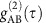 to be evaluated in four different polarization settings (AB) with a single measurement, that is, 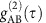, 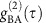, 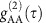, 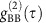 are measured simultaneously. Having four measurements for each polarization basis has two main advantages: it compensates for measurement drifts and it allows us to calculate the degree of correlation without any assumption on the polarization state of the photons. In particular, we do not need to assume, for example, that 
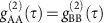
, and we can simply average the observed results, that is, 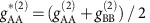. The degree of correlation is then calculated using the following formula 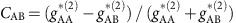. Finally, the fidelity is calculated via^5^ 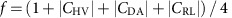.

## Additional information

**How to cite this article:** Trotta, R. *et al.* Wavelength-tunable sources of entangled photons interfaced with atomic vapours. *Nat. Commun.* 7:10375 doi: 10.1038/ncomms10375 (2016).

## Supplementary Material

Supplementary Information Supplementary Figures 1-3, Supplementary Notes 1-4 and Supplementary References.
