# Supplementary material for: Wavelength-tunable sources of entangled photons interfaced with atomic vapours
Source: Nat Commun. 2016 Jan 27;7:10375. doi: 10.1038/ncomms10375 (PMC4737804; doi:10.1038/ncomms10375)
Supplement: Supplementary Information — Supplementary Figures 1-3, Supplementary Notes 1-4 and Supplementary References. [file ncomms10375-s1.pdf]

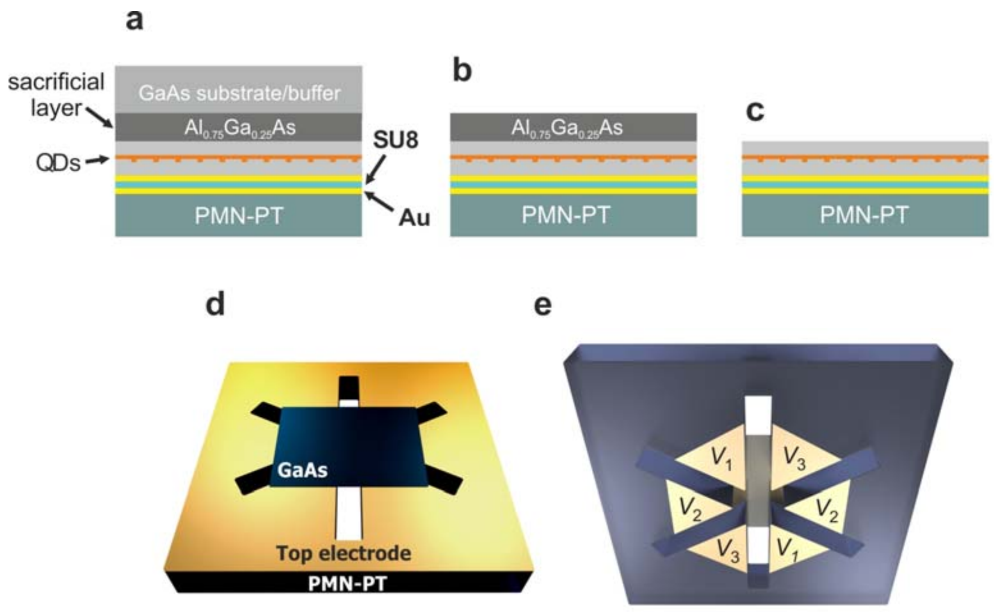

Supplementary Figure 1. Sample processing and device layout. (a)-(c). Processing steps required for the fabrication of the six-legged semiconductor-piezoelectric device used in this work. (d)-(e). Sketches of top and bottom views of the final device.

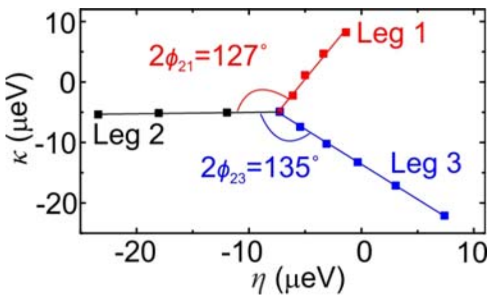

Supplementary Figure 2. Estimating the angles of the applied stresses. Behaviour of k as a function of η when the voltages across each pair of legs are swept independently from the others. The estimated angles between the stress exerted by the different legs are also reported.

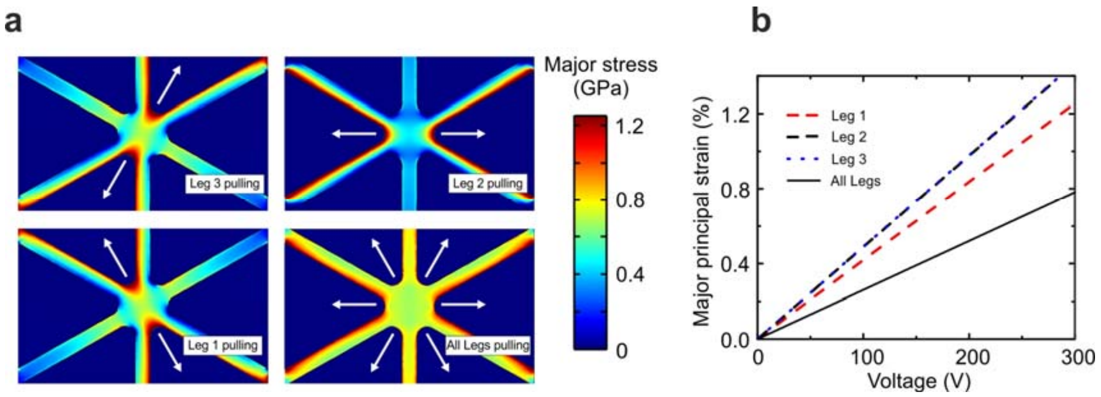

Supplementary Figure 3. Finite-element calculations. (a). Finite-element simulations of the major stress when the same voltage (200 V) is applied to the different pairs of legs. (b). Major principal strain as a function of the voltage applied to the different pairs of legs.

SUPPLEMENTARY NOTES

Supplementary Note 1

Device processing. Supplementary Figure 1 shows the processing steps required for the fabrication of the six legged device used in this work. Standard clean-room processing is used to deposit a 10/100 nm-thick Cr/Au layers on the surface of both the GaAs sample and the micro-machined PMN-PT actuator, which was processed by femtosecond laser cutting in a six-legs design (see Supplementary Figure 1d). Six metal contacts are also evaporated at the bottom of each leg, as sketched in the Supplementary Figure 1e. This allows independent voltages to be applied to the six-legs with respect to the top electrically-grounded contact. A piece of the GaAs wafer (size ~ 1 mm) is integrated onto the patterned PMN-PT via a flip chip process that involves standard epoxy-based resist (SU8) hard-baked at 250 ˚C (see Supplementary Figure 1a). Two etching steps are then used to release the GaAs nanomembrane containing the QDs from the host substrate (see Supplementary Figure 1b-c): (1) A phosphoric acid solution (H_3PO_4:H_2O_2) followed by citric acid solution are used to etch away the substrate and the buffer layer down to the $Al_{75}Ga_{25}As$ sacrificial layer. (2) The sacrificial layer is then completely removed by a HF solution, leaving the GaAs nanomembranes containing the QDs tightly bound on the piezo-legs. The whole device is then integrated onto a chip carrier with patterned electrodes (see Figure 1b in the main text).

Supplementary Note 2

Theoretical background. The behaviour of the FSS (s) and the polarization direction of the exciton emission (θ) vs. the voltages applied to the piezo-legs can be explained by a

theoretical model developed for the exciton Hamiltonian that takes into account the effect of in-plane stresses of variable magnitude, direction and anisotropy (see Ref. 1). This theoretical model can be used to demonstrate the independence of the three piezo-legs, and allows the direction of the stress they exert to be estimated, as described in the following.

Ignoring terms proportional to the identity matrix that are not relevant here, the exciton Hamiltonian under the effect of an in-plane stress (described by two principal stresses P_1 and P_2 applied along an arbitrary direction ϕ with respect to the [110] axis) can be written as:

$$H = \begin{pmatrix} \eta_0 & k_0 \\ k_0 & -\eta_0 \end{pmatrix} + \begin{pmatrix} \alpha \cdot \Delta p \cdot \cos(2\phi) & \gamma \cdot \Delta p \cdot \sin(2\phi) \\ \gamma \cdot \Delta p \cdot \sin(2\phi) & -\alpha \cdot \Delta p \cdot \cos(2\phi) \end{pmatrix} = \begin{pmatrix} \eta & k \\ k & -\eta \end{pmatrix} \tag{1}$$

where η_0 and k_0 are parameters describing a generic QD featuring structural symmetry C_1 at zero applied stress. α and γ are effective parameters related to the elastic compliance constants normalized by the valence band deformation potentials and $\Delta p = P_1 - P_2$ is the stress anisotropy. Considering that α / γ ~1 (see Ref. 1), equation 1 implies that

$$\tan(2\phi) \approx \frac{(k - k_0)}{(\eta - \eta_0)}. \tag{2}$$

On the other hand, it can be easily shown that the polarization direction of the high energy component of the exciton (θ) and the magnitude of the FSS (s) combine each other to give $\eta = s \cdot \sin(2\theta)/2$ and $k = s \cdot \cos(2\theta)/2$. Since s and θ are the observables in the experiment, it is therefore possible to calculate η and k and, via equation (2), to obtain the direction of application of the stress.

Supplementary Figure 2 shows the behaviour of k as a function of η when the voltages across each pair of legs are swept independently from the others (e.g., V_2 is swept at V_1=V_3=0). Despite each of them having a different effect on the QD parameters, all the points lie on straight lines, which intersects at η_0 and k_0 (the values of these parameters match previous theoretical calculations performed for similar QDs, see Ref. 2). By using equation (2) to estimate ϕ for each pair of legs (see the solid lines), we find that Leg 2 is indeed oriented along the [1-10] direction, while Leg 1 and Leg 3 are displaced by 63.5˚ and 67.5˚, respectively. The small deviations from the expected 60˚ are attributed to imperfections in the device fabrication and processing. The data of Supplementary Figure 2 confirm that our six-legged device is capable to deliver three independent stress fields to single QDs, the key ingredient to the implementation of an energy-tunable source of entangled photons.

Supplementary Note 3

Extracting QD linewidth. In the Figure 4b of the main text we have simulated the intensity of the QD emission line when its energy is swept across the hyperfine line of Cs. This simulation allows the linewidth of the QD emission to be estimated, as described in the following.

We begin by correcting the non-perfect calibration of our spectrometer. The spectral position of the hyperfine lines of Cs and their splitting can be indeed used as an absolute energy reference. For this reason, we subtract an offset of 458 μeV to our experimental data and we leave the linear dispersion of the spectrometer as a free parameter for the simulations (see the following). In addition, we normalize the intensity of the X emission

line so that 0 corresponds to the minima in the transmission and 1 to the mean of all the data points outside the Cs doublet.

To calculate the X intensity through Cs, we assume a Gaussian emission profile of the QD with the linewidth as free parameter, and we simulate the measured transmission spectrum of the Cs at the temperature used in the experiment (see Ref. 3 for more details about the transmission measurements). By convolving the transmission spectrum with the Gaussian function we find the best agreement to the experimental data via least-squares minimization and using the QD linewidth and the linear dispersion as the only free parameters.

Supplementary Note 4

Finite-element calculations. In the main text, we state that our device allows for full control of the in-plane stress tensor (we recall that any in-plane stress configuration can be described by two principal stresses P_1, P_2 applied at an angle ϕ with respect to the [110] direction of the GaAs crystal). To demonstrate this statement, we perform finite element (FEM) simulations taking into account the real geometry of our device, and using known parameters for sizes and elastic/piezoelectric properties for PMN-PT and GaAs[4,5]. The four panels of Supplementary Figure 3a show examples of the FEM simulations for different set of voltages applied to the 6 legs: it can be clearly seen that the major stress (P_1) axis can be rotated by 120° (because of symmetry 90° is sufficient to demonstrate full control over ϕ) with respect to the [100] direction of the GaAs matrix, with stress magnitudes as large as 1 GPa. It is worth noting that in the configuration shown in Supplementary Figure 3 – where the central gap between the piezo-legs (80 µm) is shorter than the piezo-legs length (1500 µm) – one expects strongly enhanced

strain values on the central part of the membrane due to a geometrical "focusing" effect, similar to Ref. 6. More specifically, the FEM simulations predict that it is possible to achieve biaxial strain values as high as ~ 1% (see Supplementary Figure 3b). Supplementary Figure 3 also shows that the in-plane strain transferred to the GaAs nanomembrane by the different legs is similar only when the QD is sitting in the very central region of the device. This might explain the different shifts of the X energy as a function of the voltages applied to the three pairs of legs (see the main text).

It should be noticed that the value of the strain given above cannot be directly compared with the measurements, as the PMN-PT parameters we used in the simulations are specified for room temperature, while the experiment is performed at low temperatures. In spite of the fact that at low temperature we do expect less strain for the same applied voltage, the PMN-PT parameters at 4 K are not known. However, using the known shift of the X emission line at s=0 (see Figure 2e in the main text) we can estimate[1] the maximum achieved in-plane biaxial strain to be of about 0.25%.

SUPPLEMENTARY REFERENCES

[1] Trotta, R., Martín-Sánchez, J., Daruka, I., Ortix, C. & Rastelli, A. Energy-Tunable Sources of Entangled Photons: A Viable Concept for Solid-State-Based Quantum Relays. *Phys. Rev. Lett.* **114**, 150502 (2015).

[2] Gong, M., Hofer, B., Zallo, E., Trotta, R., Luo, J.-W., Schmidt, O. G. & Zhang. C. Statistical properties of exciton fine structure splitting and polarization angles in quantum dot ensembles. *Phys. Rev.* B **89**, 205312 (2014).

[3] Wildmann, J. S., Trotta, R., Martín-Sánchez, J., Zallo, E., Schmidt, O. G. & Rastelli, A. Atomic Clouds as Spectrally-Selective and Tunable Delay Lines for Single Photons from Quantum Dots. arXiv:1505.04071 (2015).

[4] Adachi, S. GaAs, AlAs, and AlxGa1-xAs: material parameters for use in research and device applications. *J. Appl. Phys.* **58**, R1 (1985).

[5] The values of the elastic compliance constants and piezoelectric coefficients were provided by TRS Technologies (private communication).

[6] Süess, M. J., Geiger, R., Minamisawa, R. A., Schiefler, G., Frigerio, J., Chrastina, D., Isella, G., Spolenak, R., Faist, J. & Sigg, H. Analysis of enhanced light emission from highly strained germanium microbridges. *Nature Phot.* **7**, 466-472 (2013).
